# From Pen to Portal: Using Artificial Intelligence to Evaluate the Impact of Electronic Patient Information and Communication (EPIC) on the Quality of Urology Clinic Letters at a Major London Hospital

**DOI:** 10.7759/cureus.97486

**Published:** 2025-11-22

**Authors:** Theodore Patel, Nawal Khan, Jonathan Huntley, Nicholas Raison

**Affiliations:** 1 General Surgery, King's College Hospital, London, GBR; 2 Urology, King's College Hospital, London, GBR; 3 Financial Mathematics, King's College London, London, GBR; 4 School of Biomedical Engineering, King's College London, London, GBR

**Keywords:** clinic letters, epic, quality improvement, readability, urology

## Abstract

Background

High-quality documentation is an essential part of providing patients with good clinical care. Clinical letters promote continuity, reduce errors, and communicate effectively between healthcare professionals. EPIC (Electronic Patient Information and Communication), an electronic patient record system introduced across several London hospitals, aims to improve the quality and consistency of documentation. There is limited evaluation of its impact on urology clinic letters.

Aims

The main aim of this is to evaluate the impact of EPIC implementation on the quality, structure, and readability of urology outpatient letters at King’s College Hospital, a major London teaching hospital.

Methods

A retrospective analysis compared consultant-authored urology clinic letters before and after EPIC introduction. The pre-EPIC dataset included 115 patients (119 letters, 2003-2023), and the post-EPIC dataset included 170 patients (209 letters, 2024-2025). Each letter was assessed for structure, language, and readability, using objective metrics, including word and sentence counts, syllables, and recognised readability indices. Quality was further assessed using the Sheffield Assessment Instrument for Letters (SAIL).

Results

Following EPIC implementation, letters were shorter and more concise, with lower character and syllable counts. Readability improved: Flesch-Kincaid grade level decreased from 8.7 to 7.9 (p < 0.001); Gunning Fog Index decreased from 12.1 to 11.4 (p = 0.017); and Coleman-Liau Index increased from 19.0 to 20.1 (p = 0.003), indicating simpler phrasing and clearer structure. SAIL analysis showed significantly higher global quality scores (7.5 vs 6.0; p < 0.01), and improved content validity, with letters demonstrating clearer communication and better organisation.

Conclusions

The introduction of EPIC produced measurable improvements in the clarity, structure, and overall quality of urology clinic letters. EPIC enhanced documentation consistency and supported clearer communication between clinicians and patients.

## Introduction

High-quality documentation is an integral part of providing patients with good clinical care. Ensuring patients and fellow clinicians receive well-structured summaries enables better continuity of care and reduces the incidence of medical errors. Documentation can be time-consuming and often varies from clinician to clinician, depending on knowledge, clinical experience, and proficiency in the English language. Despite the importance of information in clinic letters, most doctors do not undergo any formal training or receive feedback on how well they are written. The lack of standardisation in clinical letters means that key information may not be included. Establishing standards via an electronic record system could strengthen communication and accuracy. Maintaining clear, accurate, and timely clinical records is also a professional requirement set out by the General Medical Council in Good Medical Practice [1]. 

Electronic patient record systems have replaced traditional paper documentation and revolutionised patient care through integrated data access. Patient details, including vital signs, labs, investigations, scans, and clinical reviews, can all be accessed from one system. The EPIC (Electronic Patient Information and Communication) electronic patient record system is a relatively new platform used across several hospitals in the UK. There is limited evidence evaluating the impact of EPIC on clinical letters, including readability, linguistic clarity, and structural consistency. Understanding these effects can help clinicians enhance documentation standards and improve patient outcomes. 

This study aims to evaluate the impact of implementing the EPIC electronic health record (EHR) system on the quality, structure, and readability of urology outpatient clinic letters at a major London teaching hospital. Readability, structure, and information quality were selected as outcomes because they are core features of effective clinic letters and may be influenced by EHR systems. Using artificial intelligence (AI) and natural language processing (NLP), we compared letters written before and after EPIC’s introduction to identify measurable changes in length, linguistic complexity, and document structure. The objective was to determine whether EPIC improves the clarity and consistency of clinical communication, supporting more reliable documentation and better patient care.

## Materials and methods

We conducted a retrospective study comparing urology outpatient clinic letters written before (pre-EPIC) and after (post-EPIC) the implementation of the EPIC EHR system. EPIC was implemented at Guy’s and St Thomas’ and King’s College Hospital NHS Foundation Trusts in October 2023 as part of the Apollo Programme. For the post-EPIC cohort, clinic letters were obtained from urology outpatient clinics led by consultants using the EPIC system, comprising 200 patients. For the pre-EPIC cohort, no unified clinic database existed; therefore, the most recent 200 patients seen by urology consultants and registrars were identified through the legacy EPR system. After review, 81 of these letters were written by non-consultants in different specialties and were therefore removed from this cohort.

Using the online Word Count Tools platform [2], 16 structural and linguistic variables were examined. These included total word count, characters (with and without spaces), syllable count, sentence count, unique word count (appearing once in the whole text), average word length, average sentence length, monosyllabic and polysyllabic word counts, syllables per word, and paragraph count. Difficult words (a word that does not belong to a list of 3,000 familiar words), short words (≤3 characters), and long words (≥7 characters) were also included. Readability was assessed using the Dale-Chall Readability Index [3], Automated Readability Index [4], Coleman-Liau Index [5], Flesch Reading Ease Score [6], Flesch-Kincaid Grade Level [7], and Gunning Fog Index [8]. All patient identifiers were removed to ensure confidentiality. Clinic letters were consecutively sampled to ensure representativeness. Each letter was processed using the same AI-based text analysis tool to ensure consistent measurement. Clinic letters were first analysed using an online readability tool. AI-assisted verification (GPT-4, GPT-5; OpenAI, San Francisco, CA, USA) was then used only to cross-check and validate automated readability outputs. Repeated runs (n = 4) produced minimal variation (<2% variation), confirming consistency. This study used fully anonymised clinical letters and did not involve identifiable patient information; therefore, it met NHS Health Research Authority guidance for research exemption and did not require formal ethical review. Statistical comparisons were performed using the Mann-Whitney U test (Table 1).

**Table 1:** Summary of readability indices used

Readability index	What does it measure	Score range	Interpretation of scores
Dale-Chall Readability Index (DCRI) [3]	The number of difficult/uncommon words and long sentences	4-15	4-6 = easy/primary level; 7-9 = average/adult level; >10 = difficult/academic level
Automated Readability Index (ARI) [4]	Average word and sentence length to estimate grade level	1-14+	1-6 = easy; 7-9 = plain English; 10-12 = high school; >13 = college level
Coleman-Liau Index [5]	Number of letters and sentences per 100 words	1-14+	<8 = easy; 8-12 = standard; >12 = technical
Flesch Reading Ease Score [6]	Overall reading ease based on word and sentence length	0-100	0-30 = very difficult; 30-50 = difficult; 60-70 = plain English; 90-100 = -10 very easy
Flesch-Kincaid Grade Level [7]	Converts reading ease into an equivalent school grade level	1-14+	6-8 = clear; 9-12 = moderate; >13 = complex
Gunning Fog Index [8]	Number of long sentences and complex words that appear in the text	6-20+	6-8 = simple; 9-12 = standard; 13-16 = difficult; >17 = complex

Multiple runs of the Sheffield Assessment Instrument for Letters (SAIL) were performed on all clinic letters through ChatGPT5 to confirm consistency [9]. Near-identical readability scores, with minor variations of less than 2%, were returned across three runs, demonstrating consistency of results. The SAIL tool, comprising a 20-item checklist (item-by-item correctness) and a global score (overall rating), was used to determine if clinic letters contained essential information, were well-structured, and did not include unnecessary information. As the traditional SAIL is human-scored, these results were not formally cross-validated by trained assessors, which represents a limitation of this study. However, informal comparisons were made with letters reviewed by several clinicians to ensure face validity of AI-assisted scoring. 

## Results

The post-EPIC dataset consisted of 170 patients and 209 clinic letters. These were authored by four consultants over a three-month period (October 3, 2024, to January 3, 2025), with a small number of registrar-authored letters also included. The distribution of patients per consultant was 70 (41.2%), 43 (25.3%), 31 (18.2%), and 26 (15.3%), respectively. The pre-EPIC dataset consisted of 115 patients and 119 clinic letters, spanning a 21-year period (May 8, 2003, to October 18, 2023). Of these patients, 87 (75.7%) were seen by urology consultants and 28 (24.3%) by non-urology consultants (including fellows and registrars). The short post-EPIC period makes comparison harder, as EPIC was only recently introduced, and we will repeat the analysis in the future when more data are available (Tables 2-3). 

**Table 2:** Demographics EPIC, Electronic Patient Information and Communication

	Pre-EPIC	Post-EPIC
Number of patients	115	170
Number of consultants	10	4
Number of clinic letters	119	209
Number of clinic letters by consultants	87 urology (75.7%) and 28 non-urology (24.3%)	209 urology (100%)
Number of registrars	28	0
Timeframe	21-year period (May 8, 2003-October 18, 2023)	3 months (October 3, 2024-January 3, 2025)

**Table 3:** Comparison of readability and structural metrics pre- and post-EPIC implementation (median, IQR) Data are represented as median (IQR) in pre- and post-EPIC letters. Statistical analysis was performed using the Mann-Whitney U test, and a p-value < 0.05 was considered statistically significant. p-values calculated using the Mann-Whitney U test. EPIC, Electronic Patient Information and Communication; IQR, Interquartile Range

Metric	Pre-median (IQR)	Post-median (IQR)	U-value	p-value
Words	162 (120-210)	122 (90-160)	7529	<0.001
Characters (incl. spaces)	933 (750-1120)	762 (620-900)	7529	<0.001
Characters (no spaces)	776 (630-960)	627 (520-760)	7529	<0.001
Syllables	277 (220-340)	220 (180-270)	7529	<0.001
Paragraphs	11 (8-15)	7 (5-10)	7666	0.002
Short Words (≤3 characters)	50 (42-58)	51 (43-59)	9493	0.68
Difficult Words (a word that does not belong to a list of 3000 familiar words)	51 (45-58)	51.5 (45-57)	9456	0.64
Long Words (≥7 characters)	60 (45-70)	32.5 (25-45)	7529	<0.001
Dale-Chall Readability Index	11.0 (10.5-11.6)	10.7 (10.3-11.2)	8294	0.03
Automated Readability Index	11.1 (10.4-11.8)	10.6 (9.9-11.3)	8373	0.04
Coleman-Liau Index	11.0 (10.2-11.8)	10.7 (9.9-11.4)	8437	0.05
Flesch Reading Ease Score	49.5 (47.0-52.0)	50.9 (48.0-54.0)	8580	0.08
Flesch-Kincaid Grade Level	8.7 (8.2-9.3)	7.9 (7.4-8.4)	8017	0.01
Gunning Fog Index	12.1 (11.3-12.9)	11.4 (10.8-12.0)	8294	0.03

Post-EPIC clinic letters were significantly shorter, with median word counts falling from 162 to 122 (p < 0.001), alongside reductions in character and syllable counts, indicating more concise documentation. Despite this, structural clarity was maintained, and readability indices, such as Flesch-Kincaid and Gunning Fog, showed small but consistent improvements, reflecting easier-to-read text. Language patterns demonstrated subtle shifts, with slightly longer average word length and marginally denser vocabulary, while sentence length decreased and paragraph counts increased, suggesting a more modular and organised writing style. Post-EPIC letters also contained fewer long and difficult words, indicating simpler phrasing and greater linguistic accessibility. Collectively, these findings show that EPIC implementation was associated with shorter, clearer, and more professionally consistent clinical communication, without loss of informational quality.

Most letters in both groups were written at a graduate reading level according to the Dale-Chall Readability Index Level, with 58% of post-EPIC letters and 50% of pre-EPIC letters meeting this threshold; however, results were not statistically significant (p > 0.05). Post-EPIC letters demonstrated a slightly lower Dale-Chall score (10.71 vs. 11.01), suggesting a modest improvement in readability, while the higher standard deviation in pre-EPIC letters indicated greater variability in writing style. The Automated Readability Index was also lower post-EPIC (10.6 vs. 11.2; p = 0.013), indicating slightly easier reading, and the Gunning Fog Index decreased from 12.1 to 11.4 (p = 0.017), reflecting reduced linguistic complexity. The Coleman-Liau Index increased post-EPIC (20.1 vs. 19.0; p = 0.003), suggesting that post-EPIC letters contained more key information and fewer words. The Flesch Reading Ease Score showed negligible change (49.77 vs. 49.57), keeping both groups within the “fairly difficult” category. However, the Flesch-Kincaid Grade Level decreased significantly from 8.7 to 7.9 (p < 0.001), suggesting improved accessibility and alignment with plain-language documentation guidelines. The Gunning Fog Index decreased from 12.12 in pre-EPIC letters to 11.39 post-EPIC (p = 0.017), representing a modest reduction in linguistic complexity and a shift toward clearer, more accessible clinical communication. In summary, post-EPIC letters were significantly simpler and easier to read, with lower readability scores and p-values.

The SAIL was applied to 30 pre- and post-EPIC letters to assess overall structure and content quality. Post-EPIC letters achieved significantly higher checklist scores (mean 17.8 ± 1.4 vs. 14.6 ± 2.5; p < 0.01) and global quality scores (7.5 ± 0.9 vs. 6.0 ± 1.2; p < 0.01). These results indicate that post-EPIC correspondence was clearer, more consistent, and better structured overall. However, as letters became shorter, some narrative detail was reduced, particularly in sections covering patient background, psychosocial context, and family concerns. Despite this, post-EPIC letters consistently included essential clinical information and demonstrated fewer unnecessary omissions (Table 4).

**Table 4:** SAIL metrics SAIL, Sheffield Assessment Instrument for Letters; EPIC, Electronic Patient Information and Communication

SAIL metric	Pre-EPIC (Mean ± SD)	Post-EPIC (Mean ± SD)	p-value
Checklist Score (max 20)	14.6 ± 2.5	17.8 ± 1.8	<0.01
Global Quality Score (0-10)	6.0 ± 1.2	7.5 ± 0.9	<0.01

## Discussion

Our study has shown that the most notable changes to clinic letters produced by the EPIC EHR system include a decrease in word count and long-word use, an increase in template use, and shorter reading time, while maintaining structural clarity. Post-EPIC letters were shorter yet conveyed key information with improved readability, suggesting greater efficiency. In this study, “key information” referred to the inclusion of core clinical elements - such as diagnosis, management plan, and follow-up arrangements - based on the SAIL checklist.

There is ample evidence in the literature to suggest that patients and general practitioners prefer to receive more information in outpatient clinic letters. A randomised controlled trial by O'Reilly et al. showed that although sending personalised letters to patients may not have improved their ability to recall clinical details, the letters were highly valued by patients and clinicians alike [10]. A study involving GP letters by Roberts and Partridge highlighted the importance of patients receiving clinic letters. Their findings similarly emphasised that concise, clearly structured letters improved patient comprehension [11].

There is no formal training, and there are limited templates and guidelines on how clinic letters should be written [12]. Many clinicians improve their skills with time and experience, gradually developing their clinical knowledge. The time pressure associated with seeing multiple patients in the clinic, along with producing well-written letters, can be challenging. A study by Lonergan et al. introduced several interventions - including measured readability, structure, paragraphs, and early prompts - to see if they improved outpatient urology letters. They later obtained detailed feedback from patients and found that 74% of patients found the letters easy to understand, 92% felt they were accurate to their memory of the consultation, and 72% stated that the letters helped their understanding of the visit [13].

The introduction of EHR has allowed clinicians to progress from traditional paper records to a more efficient and accessible system for storing and documenting patient information. One of the aims was to improve the quality of documentation, as well as to decrease the time spent on it, which, in turn, would improve patient care [14]. EPIC is one of the main EHR systems used globally and was rated the best overall in 2023 [15]. It is an all-in-one healthcare software system that supports clinical, administrative, and billing functions and provides both provider tools and patient access to their health information [16].

Despite the introduction of many advanced EHR systems, there is conflicting evidence about the positive impact they have had on patient care. A systematic review by Baumann et al., on the impact on clinical documentation time, involving 28 studies both pre- and post-EHR, showed an overall increase in documentation time for physicians (16% to 28%), interns (20% to 26%), and nurses (9% to 23%). However, this may be because the healthcare workers involved in the study had longer experience with paper documentation and, therefore, were naturally slower with a new electronic system. There was also an additional lack of long-term follow-up in this study [14].

There are very limited studies analysing the effects that EPIC has had on the quality of clinic letters, and this is, to our knowledge, the first study looking at the impact it has had on urology outpatient letters. We studied different variables using several scoring systems. The use of AI, with the help of word count tools, helped us further evaluate these effects. Despite this, our study was not without limitations. Although we used over 209 clinic letters, our study involved a single speciality, including urology patients only. As this was a single-centred, retrospective study within one speciality, the findings may not be generalisable to other NHS trusts or EPIC implementations; multicentre evaluation is needed to validate these results. The letters in the post-EPIC cohort were written by a total of four consultants, and the results may have been different if a higher number of consultants had been included in the study. Our study analysed quantitative metrics and median values; therefore, it may not have reflected variability across individual notes or taken into account the clinical context. Further data collection and analysis need to be carried out to determine whether these changes in clinical letters resulted in improvements in clinical care and patient understanding.

## Conclusions

Implementation of the EPIC EHR system at a major London hospital produced measurable changes in urology outpatient clinic letters. Post-EPIC letters were significantly shorter, with fewer characters, syllables, and long or unique words, yet contained more paragraphs and sentences, creating a more modular structure. Readability metrics showed modest improvements, including lower Automated Readability and Gunning Fog scores and a reduced Flesch-Kincaid Grade Level, indicating slightly greater accessibility. However, most letters still fell within the “fairly difficult” range, reflecting the persistence of technical terminology. These results suggest that EPIC can promote greater consistency and concision in clinical correspondence, likely aided by templates and standardised formats. Further optimisation, such as plain-language initiatives and patient-focused content, could enhance accessibility without compromising clinical detail. This would thereby support clearer communication between healthcare professionals and patients.
